# A Modular Mobile Health App for Personalized Rehabilitation Throughout the Breast Cancer Care Continuum: Development Study

**DOI:** 10.2196/23304

**Published:** 2021-04-13

**Authors:** Ji Young Lim, Jong Kwang Kim, Yoon Kim, So-Yeon Ahn, Jonghan Yu, Ji Hye Hwang

**Affiliations:** 1 Department of Physical Therapy General Graduate School of Medical Sciences Konyang University Daejeon Republic of Korea; 2 Medi Plus Solution Seoul Republic of Korea; 3 Department of Rehabilitation Medicine National Rehabilitation Center Seoul Republic of Korea; 4 Kangbuk Samsung Hospital Seoul Republic of Korea; 5 Division of Breast Surgery, Department of Surgery Samsung Medical Center Sungkyunkwan University School of Medicine Seoul Republic of Korea; 6 Department of Physical and Rehabilitation Medicine Samsung Medical Center Sungkyunkwan University School of Medicine Seoul Republic of Korea

**Keywords:** breast cancer, mobile health, rehabilitation, cancer continuum

## Abstract

**Background:**

Although many mobile health (mHealth) apps have evolved as support tools for self-management of breast cancer, limited studies have developed a comprehensive app and described the algorithms for personalized rehabilitation throughout the breast cancer care continuum.

**Objective:**

This study aimed to develop a comprehensive mobile app and to describe an algorithm that adjusts personalized content to facilitate self-management throughout the breast cancer care continuum.

**Methods:**

The development process of the modular mHealth app included the following 4 steps: (1) organizing expert teams, (2) defining evidence-based fundamental content and modules, (3) classifying user information for algorithms to personalize the content, and (4) creating the app platform and connectivity to digital health care devices.

**Results:**

We developed a modular mHealth app service, which took 18 months, including a review of related literature and guidelines and the development of the app and connectivity to digital health care devices. A total of 11 functionalities were defined in the app with weekly analysis. The user information classification was formulated for personalized rehabilitation according to 5 key criteria: general user information, breast operation type, lymph node surgery type, chemotherapy and hormonal therapy use, and change in treatment after surgery. The main modules for personalized content included a self-monitoring screen, personalized health information, personalized exercise, and diet management.

**Conclusions:**

The strength of this study was the development of a comprehensive mHealth app and algorithms to adjust content based on user medical information for personalized rehabilitation during the breast cancer care continuum.

## Introduction

### Background

Breast cancer is the most frequently diagnosed cancer among women worldwide and in Korea. In 2019, there were an estimated 24,010 cases, suggesting an increasing trend [1]. Patients with breast cancer undergo a long course of diagnostic, treatment, and posttreatment procedures; long-term survivorship; and end-of-life stages [2]. Different treatments for breast cancer result in a series of treatment-related problems such as upper limb dysfunction, fatigue, and sleep disturbance [3-6], and these side effects induce short-term, long-term, and late effects [7]. Patients with breast cancer should be educated regarding self-management of their physical symptoms and functions and changing their behavior to promote a better quality of life [4,8]. Therefore, an accessible system is essential for the resolution of symptoms and health care concerns across the breast cancer care continuum.

From this viewpoint, mobile health (mHealth) apps in cancer care have emerged as an attractive technology for enabling symptom and disease management and promoting healthy lifestyles such as increasing physical activity [9-13]. An increasing number of mHealth apps for patients with breast cancer are being developed; these apps aid in establishing an association between patients and health care professionals [9,14-16]. Currently, approximately 600 mobile apps for patients with breast cancer are available in the iOS and Android markets [16]. The potential advantages of providing interventions using mHealth apps include ease of use, cost-and resource-effectiveness, and personalization during treatment [17]. It has positive effects on physical activity, weight loss, quality of life, functional fitness, and psychological factors (such as anxiety, depression, and distress) [18-22].

As the need for tailored intervention with mobile apps and wearable trackers among patients with breast cancer has increased [17,23], adjustment of the cancer treatment plan by considering the characteristics of individual patients has become important. However, a recent systematic review [9] revealed that most mobile apps focus on only providing relevant information or recording side effects during the treatment of breast cancer rather than providing personalized rehabilitation [22,24-26].

### Objective

To the best of our knowledge, no studies have specifically focused on developing a comprehensive mHealth app for providing personalized content during the breast cancer care continuum, although several studies have examined the feasibility or effects of interventions that use an app during treatment. Therefore, we developed a modular mHealth app that can be personalized according to surgery type and treatment modality during the breast cancer care continuum. This study aimed to describe algorithms for personalizing the content of the app to support self-management and address the development process.

## Methods

### Development Process

#### Stage 1: Expert Team Organization for App Development

For app development, an expert team was organized, which was comprised of a multidisciplinary research team and development team. The research team included a breast surgeon, 2 physical and rehabilitation medicine physicians, 2 physical therapists, 2 exercise experts, and 2 nutritionists. The development team consisted of app developers and service programmers. The development team (2 professional developers and 2 service planners) had experience in developing an mHealth app for chronic disease management, pregnancy, and patients with stomach, colon, and prostate cancers. The app design team included 2 designers who had extensive expertise in designing applications for cancer patients.

#### Stage 2: Evidence-Based Fundamental Content and Modules

This app was developed for Korean women with breast cancer, and it aimed to promote health care from the postoperative period to the end of treatment. To clarify the actual content and modules of the app for breast cancer, the research team exchanged mutual opinions through regular meetings and sorted the content. The content and modules were included after sufficient discussion and review of the relevant research experience. The final decisions on the app’s content and modules were determined by a consensus of all experts. A total of 11 functionalities was included in this app to help users manage the side effects of their diseases and treatments, comorbidities, and lifestyle choices with a weekly analysis function (Table 1).

**Table 1:** Functionality and key characteristics of the mobile app.

Functionality	Key characteristics (evidence-based)
Expert consultation	Provides consultations related to exercise and nutrition using text messages, voice recordings, and images; health care professionals respond to the questions within 24 hours.
Self-monitoring (today’s to-do list)	Allows users to self-check their treatment-related symptoms; allows users to self-assess their physical activity, calorie consumption, sleep, and stress information.
Personalized health information and education	Offers health information and education on exercise, nutrition, and disease (updated every Monday in webzine form); offers health information and education according to surgery, treatment type, side effects, and comorbidities.
Personalized exercise management	Offers aerobic exercise with the goal of a specific heart rate and exercise time according to the user’s health information; offers an arm and shoulder exercise program with a video consisting of four steps according to the user’s information (surgery and treatment type) and exercise journal.
Physical activity management	Recommends target step intensity (normal, brisk, and run), calories, heart rate, and step count (eg, 5000 steps and 200 kcal).
Diet management	Provides daily guidelines for each food group: Users can record the foods ingested during breakfast, lunch, dinner, and refreshments for a day using speech recognition and text input; recommends nutritional intake according to comorbidities.
Sleep management	Allows users to track total sleep times, sleep efficacy, REM^a^ sleep time, NREM^b^ sleep time, and sleep quality using a smart band.
Comorbidity management (weight, blood pressure, blood glucose)	Allows users to use a journal format and Bluetooth-enabled smart device: The target blood pressure and blood glucose levels are indicated; allows users to track their weight, blood pressure, and blood glucose level.
Stress management	Allows users to track their stress level (categorized as good, normal, low, and high) based on heart rate variation analysis.
Medication and smoking management	Provides an alarm sound at the time when users should take their medications, allowing them to track their medication intakes; provides an alarm sound to prompt users to record their daily smoking status.

^a^REM: rapid eye movement.

^b^NREM: non-rapid eye movement.

To offer evidence-based information, the following resources were used for the app: the American College of Sports Medicine Exercise Prescription Guidelines for Cancer Survivorship; exercise program for patients with breast cancer [8,27-29]; neurophysiological mechanisms of sleep [30-34]; Korean Society for the Study of Obesity guidelines [34,35]; Korean Society of Hypertension guidelines [36]; and Korean Diabetes Association guidelines [37].

#### Stage 3: Classification of User Information for the Algorithms for Personalized Content

**Figure 1:**
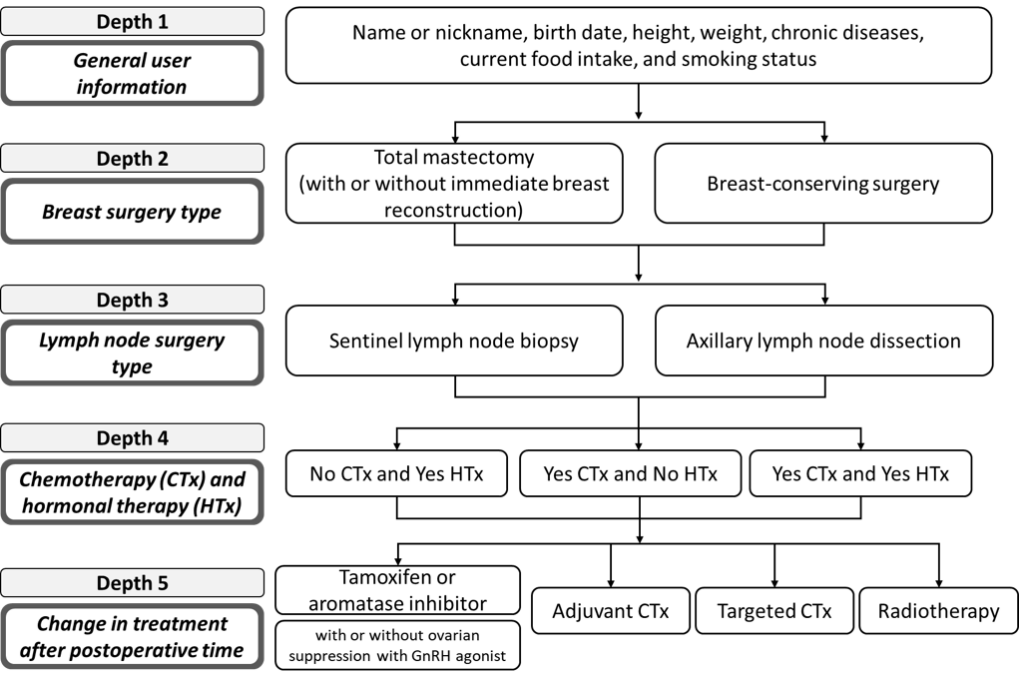
Classification system of user information for the algorithms for personalized content.

To provide personalized content, we discussed and sorted the representative treatment course after surgery for breast cancer according to the aim and scope of the app. The following information was included in the algorithm for personalized content (Figure 1): general user information, breast surgery type, axillary surgery type, treatment using chemotherapy and hormonal therapy, and treatment change after postoperative period (total of 5 years). This classification complied with the National Comprehensive Cancer Network Guidelines for patients with breast cancer [38] and reflected the treatment process of breast cancer patients in Korea. At the end of the review and discussion, the content was finally classified into a total of 34 treatment courses.

General user information included the following: name or nickname, sex, birth date, height, weight, chronic diseases (eg, hypertension, diabetes, and hyperlipidemia), current food intake (eg, vegetables, fruit, fish, meat, and rice), and smoking status. To utilize personalized content, users input breast surgery type, lymph node surgery type, surgery date, hospital discharge date, and current treatment (eg, chemotherapy, radiation therapy, and hormonal therapy), including general user information.

#### Stage 4: Creation of the Modular mHealth App Platform and Connectivity to Digital Health Care Devices

This app was developed for use on both Android and iOS platforms and can transfer data via connection with Bluetooth-enabled wearable smart devices. Health care professionals can use a web-based open architecture management (OAM) program to monitor all data, including those on app service use and wearable or smart devices.

The wearable device includes a DoFit smart band worn on the wrist (NF-B20, Medi Plus Solution, Seoul, South Korea), which allows the measurement of physical activity, stress level, heart rate, and sleep information through a built-in 6-axis accelerometer, gyroscope, and photoplethysmography sensor. Energy expenditure and physical activity intensities were analyzed on the basis of heart rates and step counts. Stress level is provided on the basis of heart rate variation analysis of heart rates collected during daily activities. Moreover, the smart device linked to the app includes a blood pressure gauge (UA-651BLEm, A&D Medical, Tokyo, Japan), glucose monitor (GM01AAB, i-SENS, Seoul, Korea), and scale (XMTZC04HM, Xiaomi Corporation, Beijing, China). The Korean certification test verified the appropriate level of radiation exposure from health care devices.

In order to use data from this platform and digital health device and for use of personal information and information from mobile and wearable devices, consent is obtained while joining this service. Data generated from the platform are protected through protection of personal information and storage of data in our service platform server after deidentification of the data.

## Results

**Figure 2:**
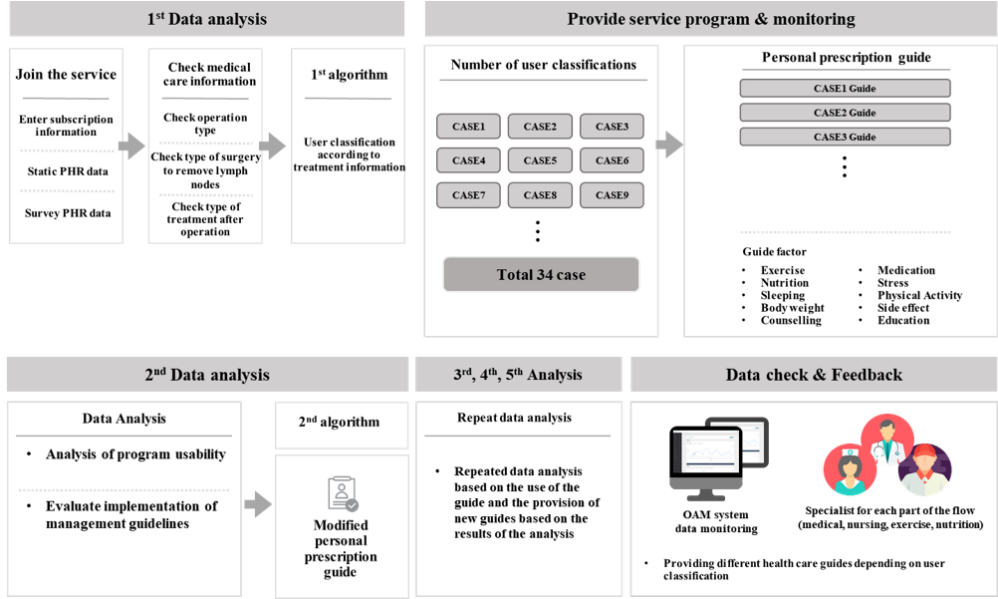
Service flowchart of the development of the modular mobile health (mHealth) app for personalized rehabilitation. OAM: open architecture management; PHR: personal health record.

We required 18 months to review the database, related literature, and guidelines as well as to develop and launch the 11 functionalities, connectivity with a digital health care device, monitoring program, and app testing. Figure 2 presents the service flow of the app. The overall service flow of the developed app is as follows: The user creates an account (subscription information and personal health record data) and enters medical care information (breast surgery type, type of surgery to remove lymph nodes, and postoperative treatment). On the basis of the treatment information, the first algorithm is applied, and the user case is classified. Personalized guides are provided for exercise, side effect education, and nutrition. The second algorithm analyzes the program usability and whether the implementation follows management guidelines and then personalizes the guide. By interworking with health care devices such as smart bands and the establishment of the OAM system for data monitoring, the service platform enables patient-health care expert communication.

### Key Modules for Personalized Content Based on Classification of User Information

**Figure 3:**
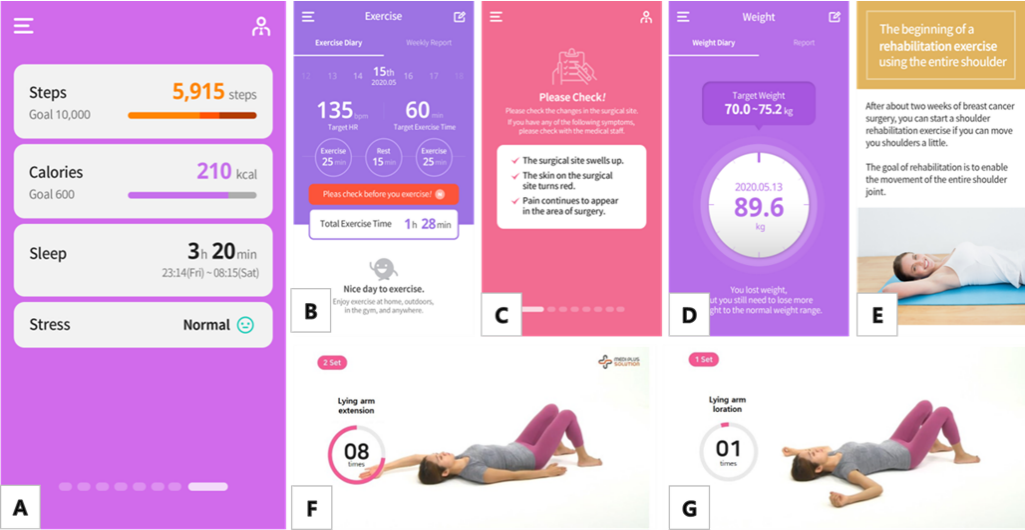
Screenshots of the representative app functionality: (A) self-monitoring, (B) exercise management, (C) main (today’s to-do list), (D) weight management, (E) personalized education and information, and (F) arm and (G) shoulder exercises.

Figure 3 shows screenshots representing the app’s functionality. According to the classification of user information algorithms for personalized content, if a user enters their information, the app provides personalized content and goals on the self-monitoring screen, including personalized health information, aerobic exercise, arm and shoulder exercises, and nutrition management.

### Self-Monitoring Screen (Today’s To-Do List)

Once the user information is entered, the self-monitoring components are displayed by priority at login. The treatment-related symptom checklist is displayed as treatment changes occur. For example, if a patient with breast cancer initiates chemotherapy, medical caution related to side effects is shown on the main screen, and users are prompted to check their health conditions when they log in. After the treatment is completed, the monitoring screen changes according to the user’s comorbidities. If the user has a chronic disease, the management screen is displayed as a priority. On the self-monitoring screen, the last screen displays a brief graph showing step count, calories, sleep details, and stress level, regardless of health status.

### Personalized Health Information

Information on health condition, managing side effects, and medical precautions are displayed in 3 categories (disease, nutrition, and exercise). The information is adjusted according to user health information such as surgery date, treatment type, and chronic disease and updated every Monday. From 1 to 5 weeks postoperative, the user is offered relevant management information. If users input treatment information such as chemotherapy, radiotherapy, and hormonal therapy, this functionality will describe the side effects, nutrition, and exercises needed during treatment. Chronic disease and wellness management information is provided after the treatment is completed.

### Personalized Exercise

#### Arm and Shoulder Exercise Program

The exercise program was developed by rehabilitation experts based on the aforementioned studies in stage 2. This home-based video exercise program consists of 4 stages that gradually progress from active-assisted to arm strengthening exercises.

**Figure 4:**
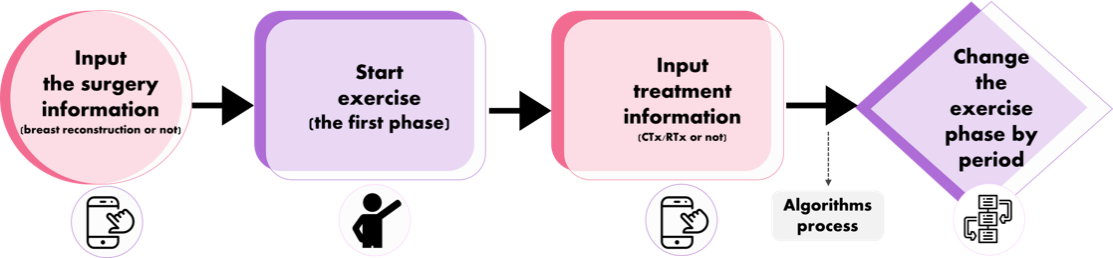
Process of applying the personalized arm and shoulder exercise program. CTx: chemotherapy; RTx: radiation therapy.

The rehabilitation experts designed algorithms specific to surgery and treatment types. The changes occur automatically, and the start time of each stage differs according to surgery and treatment type. These details include immediate breast reconstruction, chemotherapy, and radiotherapy. The process of applying the algorithm for the shoulder and arm exercise program is shown in Figure 4.

#### Aerobic Exercise for Breast Cancer

According to the user’s treatment information, the target heart rate and exercise time are automatically adjusted in accordance with the American College of Sports Medicine guidelines [39]. The initial baseline is set according to the treatment information that the user inputs at sign-up. As the week progresses, an algorithm determines the heart rate goal and gradually increases the exercise time. For instance, when chemotherapy information is entered, the target heart rate in this stage is immediately changed to control the exercise intensity; the same change occurs when chronic disease information is entered.

Depending on the estimated rate of perceived exertion after exercise termination, the exercise goal and duration are increased, decreased, or maintained. If the user records “hard” more than twice per week or “very hard” only once per week, the exercise phase is lowered. In addition, heart rate is measured in real time using a smart band during aerobic exercise. If the user’s heart rate exceeds the target, an alarm will sound to notify the need to adjust the exercise intensity.

### Diet Management

The algorithm for calorie intake is calculated using the original intake per food group, body mass index, and age that the user inputs at sign-up. This module recommends a meal plan, including food groups and intake based on the Korean diet. Furthermore, users can check whether they are consuming well-balanced food types and amounts through a graph that indicates “lacking” in gray, “appropriate” in green, and “excessive” in red. At the bottom of the diet management screen, the recommended nutrition plan changes depending on the presence or absence of chronic diseases. For instance, consumption of protein and fat is displayed for users who have undergone surgery from week 1 to 5 and who have no chronic diseases. If the user has diabetes, essential nutrients are adjusted to include carbohydrates and fat. This functionality allows users to compare their intake and recommended dietary allowance. There are restrictions on adjusting diet plans depending on the user's dietary preferences (eg, vegetarian) or taboo foods (eg, religious diet).

## Discussion

### Principal Findings

In this study, we documented the process of developing a modular mHealth app to provide personalized content based on surgery type, treatment process, and chronic disease(s). This study specified the algorithm used to modify the content by surgery and treatment types and according to the analysis of user health records. In addition, to improve the motivation and benefits of using the mHealth app [40,41], we used assistant technology (ie, smart band) and professional support for monitoring and providing real-time feedback (ie, OAM program) during app use.

The key feature of the developed app is that it is not limited to specific treatment groups such as those treated with chemotherapy or radiotherapy and is a comprehensive self-management tool available during the breast cancer care continuum. To cover the characteristics of diverse users and provide personalized content, 34 user cases were classified according to surgery information and treatment process. A previous research study [41] developed an information-centered app that consisted of 5 modules according to the information needs of women with breast cancer during treatment based on interviews and discussion with experts. They included 8 information items — stages of diagnosis, adjuvant chemotherapy, operation, chemotherapy, radiation therapy, endocrine therapy, targeted therapy, and rehabilitation. However, the focus of our study was not simply providing information but providing personalized content with a comprehensive self-management tool during the breast cancer care continuum according to the different surgery types, treatments, and chronic diseases.

With regard to the development of a comprehensive app, most modules were consistent with those from previous research [42] that provided the framework, which consisted of 8 themes, including information related to treatment, physical activity (exercise and rehabilitation), emotions (mental support, music therapy, and sufficient sleep), diet, health records, social resources, experience sharing, and expert consulting. The aforementioned study investigated the needs information through focus group interviews with women who completed different therapies or received long-term hormonal therapies. In this study, according to a review of a previous study and discussions with a multidisciplinary team, 11 modules were developed covering health information and education, self-monitoring, exercise and physical activity management, diet, sleep, comorbidity, stress, medication and smoking management, and expert consultations. Particularly, our study differs from previous studies in that the content of some modules in this developed app was adjusted primarily according to the user's characteristics (eg, type of surgery and treatment) and can be changed according to comorbidities and diseases. Some discrepancies between our results and those of previous studies may be due to differences in culture, development approach, and target user.

### Limitations

Although this study aimed to develop an app that offers comprehensive and personalized health management content during the breast cancer care continuum, some concerns need to be addressed at a later stage. This app has limitations in adjusting content based on the factors that need to be considered for highly personalized mHealth interventions such as symptom burden, weather, and treatment cycle. To date, electronic medical record data have not been directly linked with the app. To obtain personalized content, users must update their health and treatment information directly in the app. If this concern is resolved, it may be beneficial for users to use the app. Moreover, long-term engagement with and adherence to the comprehensive mHealth-supported intervention must be investigated, and additional analysis of needs and satisfaction according to user characteristics will be required.

Despite these limitations, this is an important first step in realizing a comprehensive and personalized, rehabilitation-based mHealth app for patients with breast cancer during the breast cancer care continuum.

### Conclusion

This app was developed to facilitate the comprehensive and personalized rehabilitation of patients with breast cancer throughout the treatment course using algorithms to deliver personalized content and change the user information accordingly and to motivate and monitor patients using a digital health care device that tracks the user’s information and communication with health care experts. This study enlisted algorithms to provide personalized content and describe the functionality of the comprehensive app for patients with breast cancer. In the future, its efficacy and clinical effectiveness as a management solution should be evaluated through clinical management, continuous upgrades based on the latest guidelines, and user feedback.

## Abbreviations

mHealth: mobile health
OAM: open architecture management
